# Periodization and Programming in Sports

**DOI:** 10.3390/sports9020013

**Published:** 2021-01-20

**Authors:** James P. Fisher, Robert Csapo

**Affiliations:** 1 Faculty of Sport, Health and Social Sciences, Solent University, E Park Terrace, Southampton SO14 0YN, UK; james.fisher@solent.ac.uk; 2 Strength and Conditioning Society, Via del Fontanile Anagnino 159, 00118 Rome, Italy; 3 Research Unit for Orthopaedic Sports Medicine and Injury Prevention, ISAG, UMIT Tirol, Eduard-Wallnöfer-Zentrum 1, 6060 Hall, Austria

Periodization is a generally accepted approach to manage athletic performance by the sub-division of training programs into sequential, specifically focused training periods. Periodization implements structured variability into the training process, with the aim of maximizing performance in the most critical phases of the competitive season and/or improving long-term development. A recent review specifically compared periodized and non-periodized resistance training practices for strength and hypertrophy adaptations [1]. This prompted a series of responses considering the SAID principle (specific adaptations to imposed demands) and the use of heavier-loads close to testing time-points [2], and, often overlooked, confounding variables which appear to impact strength and hypertrophy adaptations (e.g., exercise selection, supervision, etc.) [3].

The construct of periodization in strength and conditioning for athletes appears to originate from the general adaptation syndrome (GAS) proposed by Hans Selye [4]. However, Selye’s original work was based on a series of rodent studies testing the stress response to sub-lethal doses of different drugs (e.g., morphine, atropine), and stimuli (e.g., temperature, exercise, etc.), so results may not be readily applicable to the field of strength and conditioning [5]. In debate, the GAS model has been defended in its framework for periodization for the management of stress and fatigue to adaptation during sports training [6]. Whilst the views of the Strength and Conditioning Society (SCS) are to support and promote effective methods in enhancing athletic performance, of which periodization is a generally accepted practice, it is also an academic organization priding itself in the search for the scientific truth in exercise physiology, health, and human performance. As such, Drs. Jeremy Loenneke and Greg Haff presented a point: counterpoint discussion of periodization at the 2019 Strength and Conditioning Society conference in Madrid. This special issue stems from this interesting and exciting debate and pursues the aim to collate scientific evidence relating to periodization and programming in sports.

Within this special issue we have published articles concerning male and female elite soccer players [7,8], more specifically relating to how the use of detraining periods [7], and seasonal transitions and coaching influence [8] affect physiological performance markers. In addition, there are publications looking at the extension of nonlinearity programming into periodization [9]—certainly a topical as to how programming and periodization relate. Further publications include consideration of strength and power adaptations resulting from block periodization across persons of different training status [10], and training organization in bodybuilders [11]. To conclude, the final contributions to this special issue include a review of tapering and peaking practices for maximal strength adaptations for powerlifting performance [12], the effects of concurrent training and prolonged neuromuscular performance even after cessation of explosive strength training [13], and lastly, power profiling of professional U23 cyclists through periods of a competitive season—notable for the large sample size in this calibre of athlete [14].

Importantly, all the published articles within this special issue provide sensible, evidence-based information that practitioners and athletes can use to inform their own methods. Furthermore, there is important application to the layperson looking to improve their strength, health, and wellbeing.

As guest editors, and on behalf of the Strength and Conditioning Society (SCS), we would like to thank the editorial staff at *Sports* for supporting this special issue, as well as the authors for their submissions, and the reviewers for their time and commitment to academic and scientific progress—without peer-review, these publications would not be possible.
